# Strontium doping of bone graft extender: Effect on fixation of allografted experimental implants

**DOI:** 10.3109/17453674.2011.618909

**Published:** 2011-11-24

**Authors:** Marianne T Vestermark, Ellen-Margrethe Hauge, Kjeld Soballe, Joan E Bechtold, Thomas Jakobsen, Jorgen Baas

**Affiliations:** ^1^Orthopaedic Research Laboratory, Department of Orthopaedic Surgery, Aarhus University Hospital, Aarhus, Denmark; ^2^Research Unit for Rheumatology and Bone Biology, Aarhus University Hospital, Aarhus, Denmark; ^3^Orthopedic Biomechanics Lab, Excelen Center for Bone and Joint Research and Education and Minneapolis Medical Research Foundations, Minneapolis, MN, USA

## Abstract

**Background and purpose:**

Allografts are often used during revision hip replacement surgery for stabilization of the implant. Resorption of the allograft may exceed new bone formation, and instability of the prosthesis can develop. We investigated whether strontium could regulate the imbalance of fast resorption of allograft and slower formation of new bone, because it is both an anabolic and an anticatabolic agent.

**Method:**

Strontium was added to the implant interface environment by doping a hydroxyapatite bone graft extender. 10 dogs each received 2 experimental titanium implants. The implants were inserted within a 2.7-mm concentric gap in cancellous bone. The gap was filled with 50% (v/v) allograft mixed with 50% bone graft extender. The extender either had 5% strontium doping (SrHA) or was undoped (HA). After 4 weeks, osseointegration and mechanical fixation were evaluated by histomorphometry and by push-out test.

**Results:**

SrHA bone graft extender induced a 1.2-fold increase in volume of new bone, a 1.2-fold increase in allograft remaining in the gap, and a 1.4-fold increase in surface area of the bone graft extender material in contact with new bone compared to HA bone graft extender. All these increases were statistically significant. SrHA bone graft extender did not significantly improve ongrowth of bone onto the implants or improve any of the mechanical push-out parameters compared to HA bone graft extender.

**Interpretation:**

Doping of the HA bone graft extender with 5% strontium increased gap healing, preserved more of the allograft in the gap, and increased the ongrowth of bone onto the bone graft extender material, but did not improve mechanical fixation.

Due to longer life expectancy and younger ages at index operation, up to one fifth of hip replacement surgeries are now revisions. The main indication for revision is painful aseptic loosening of the prosthesis (Pedersen et al. 2005), during which periprosthetic bone loss frequently occurs. Thus, in revision surgery allografts are often used to stabilize the prosthesis. However, imbalanced rapid allograft resorption and slower formation of new bone (for mechanically securing the implant by osseointegration) have been found experimentally and may result in instability of allografted implants (Burchardt 1983, Jakobsen et al. 2010). This imbalance may partly explain the higher re-revision rate for allografted uncemented acetabular components compared to non-allografted components (Lie et al. 2004). Reduction of this imbalance may improve the outcome of allografted revision hip prostheses. Strontium is a candidate for this process, because it has both anabolic and anticatabolic effects in bone (Marie et al. 1993). Furthermore, synthetic strontium hydroxyapatite is highly biocompatible and is believed to promote bone ingrowth (Xue et al. 2007, Tian et al. 2009).

We compared strontium-doped hydroxyapatite (SrHA) with hydroxyapatite (HA) as a bone graft extender (BGE). Our purpose was 2-fold. The first was to add a local anabolic and anticatabolic effect, which strontium is thought to provide. The second purpose was to maintain graft material for longer time; thus, the allograft was mixed with BGE, because the BGE is slowly or even not at all resorbed (Hannink et al. 2007, Fellah et al. 2008). The main hypothesis was that strontium doping of the BGE would improve fixation of grafted implants, both histologically and mechanically.

## Methods and materials

**Figure 1.**
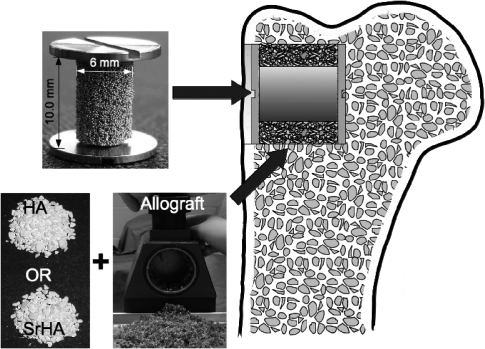
Position of the cylindrical implant with mounted end-screws in the proximal humerus with a surrounding 2.7-mm gap filled with allograft mixed with HA or SrHA.

We tested our hypothesis in a paired study in 10 dogs. The 2 treatment arms consisted of: (1) allograft mixed with strontium-doped hydroxyapatite (SrHA) BGE, and (2) allograft mixed with hydroxyapatite (HA) BGE. The graft mixtures surrounded the implants in cancellous bone of the metaphyseal part of the proximal humerus (Figure 1).

### Implants

The experimental implants were a 10-mm high cylinder consisting of a core and a coating made of ASTM-136 (Ti6Al4V) titanium (Ti) alloy. The diameter of the cores was 4.4 mm and the final mean diameter after coating was 5.7 (SD 0.2) mm. The coating was a commercially available bead-sintered porous Ti coating, donated by DePuy Inc. (Figure 1). Ti end-screws, 11.0 mm in diameter, were attached to the top and bottom of the implant and ensured a concentric 2.7-mm gap around the implant. The overall length of the implant with mounted screws was 12.0 mm.

### Bone graft extender

A precipitate of calcium-strontium hydroxyapatite was used as an experimental bone graft extender. It was prepared as follows.

Solid crystals of calcium HA with 5% calcium substituted by strontium were prepared by the method of Kumta et al. (2005) with minor modifications to the synthesis. The synthesis was performed according to the reaction equation:


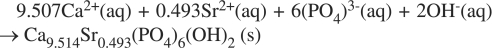


**Figure 2.**
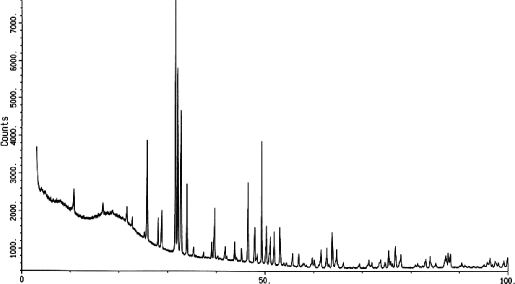
Strontium content of 4.93% for SrHA was determined by X-ray power diffraction (XRPD).

In the first 2-L beaker, 190 g of trisodium phosphate (Na_3_PO_4_) (CAS 7601-54-9; Sigma-Aldrich cat. no. 342483, batch 03621 HE) was partially dissolved in 1 L of Millipore water. After addition of 15.5 g of NaOH (CAS 1310-73-2; Sigma-Aldrich cat. no. 22146-5, lot 540948-277), the suspension was gently heated to 50°C until all the material was dissolved. In the second 2-L beaker, 268 g of calcium chloride dihydrate (CAS 10035-04-8; Fluka lot 1160484, filling code 50305149) and 30.4 g of strontium chloride hexahydrate (CAS 10025-70-4; Fluka lot 1094650, filling code 53404170) were dissolved in 1 L Millipore water. The solution in the first beaker containing trisodium phosphate and NaOH was transferred to a 5-L beaker with a stirrer, and the contents of the second beaker were slowly added with vigorous stirring for 6 h. A small-grained white precipitate was then formed. The solution was centrifuged for 5 min at 2,000 rpm and the precipitate flushed 5 times with Millipore water. The powder thus obtained was transferred to an aluminium beaker and heated to 1,000°C by increasing the temperature by 200°C per hour, and this temperature was maintained for 10 h. The oven was allowed to cool to room temperature over 12 h. The powder was kept sterile while it was ground in a mortar, weighed, and separated into batches of granules of 0.6–2 mm in diameter, which were packed individually in portions of 1.0 mL in double sterile containers ready for surgery. The yield was 92.6%, and by using X-ray powder diffraction (Diffractometer Huber G670; HUBER Diffraktionstechnik GmbH & Co. KG, Rimsting, Germany; 1.54060 wavelength, 1 h exposure time), the hydroxyapatite structure was confirmed (Figure 2). By inductively-coupled plasma mass spectrometry, the strontium content was determined to be 4.93%. The procedure was repeated for production of HA powder without adding SrCl (Table 1).

**Table 1.** The chemical formulae of SrHA and HA

SrHA	Ca_9.507_Sr_0.493_(PO_4_)_6_(OH)_2_
HA	Ca_10_(PO_4_)_6_(OH)_2_

The bone graft extenders were produced and donated by Osteologix ApS, Copenhagen, Denmark.

### Allograft

2 weeks before surgery, bones for bone graft were harvested under sterile conditions from 2 dogs not included in the study, and stored at –80°C. The proximal humerus and the distal femurs were used. All soft tissue and cartilage was removed and the bones were morselized with a Biomet bone mill, creating bone chips of 1–2 mm in size. The chips from different bones in different dogs were mixed together, portioned in sterile double containers, and refrozen at –80°C. The bone graft was thawed immediately before surgery. At surgery, the impacted allograft was divided into two 1-mL portions in a standardized container.

### Animals

We used 10 female, skeletally mature American foxhounds (mean age 12.5 (SD 1.2) months and mean weight 25.1 (SD 1) kg). The number of dogs required in this study was estimated from the formula for paired studies with normally distributed data. Based on previous studies, we expected an average coefficient of variation (CV)% ((SD / mean) × 100%) of 30% for 3 main variables: ultimate shear strength, gap healing by bone, and implant-bone ongrowth (Baas et al. 2008). We wanted to be able to detect an improvement in implant fixation between the groups of at least 30%. Based on these assumptions, 10 animals were included.

Positioning of the implants for each treatment arm was alternated systematically with a random start regarding the right and left humerus. The NIH guidelines for care and use of laboratory animals were observed, and the study was performed in our AAALAC-approved animal care facility. Minneapolis Medical Research Foundation, and the Animal Care and Use Committee approved the protocol of the study.

### Surgical procedure

Surgery was conducted under aseptic conditions with general anesthesia (initiated with intravenous thiopental (5%) and maintained with isoflurane gas (1.5%)). A 7-cm skin incision was made with cautery on the lateral proximal humerus, and the deltoid muscle was bluntly dissected to expose the proximal part of the humerus. A 2.5-mm guide wire was inserted anterolaterally at the level of the greater tubercle and oriented perpendicular to the surface. Over the guide wire, a cannulated drill (11.0-mm diameter) was used to drill a 12-mm deep cylindrical cavity at a maximum speed of 2 rotations per second. The edge of the hole was trimmed with a scalpel to remove periosteum, and the cavity was irrigated with 10 mL saline for removal of bone chips.

An implant with a mounted bottom screw was inserted into the cavity. A graft mixture of standardized volume (1 mL allograft and 1mL SrHA or HA) was tightly packed around the implant in the 2.7-mm gap and the top screw was mounted. When hemostasis was ensured, the soft tissue was closed in layers. The procedure was repeated for the contralateral humerus using the other bone graft mixture.

The dogs were given ceftriaxone (1 g i.v) and buprenorphine hydrochloride (0.0075 mg/kg/day i.m) immediately before and for 3 days after surgery.

The observation period of 4 weeks was uneventful. All the animals were fully mobilized within 2–3 days, and there were no clinical signs of infection at any point.

After 4 weeks, the dogs were sedated and killed with an overdose of hypersaturated barbiturate.

### Preparation of specimens

**Figure 3.**
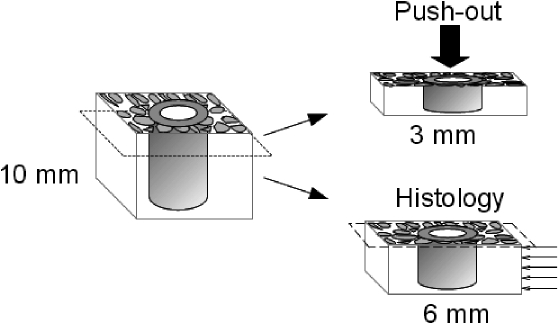
Preparation of the specimens and the transverse cutting method for the histomorphometrical analysis.

The proximal humeri were retrieved en bloc and, immediately after explantation, were frozen and stored at –20°C. Using a water-cooled-wheel diamond saw, the bone/implant specimen blocks were cut into 2 transverse blocks: a 3-mm thick block of the outermost part of the specimen block was cut and returned to the freezer for mechanical testing and a 6-mm thick block of the innermost part of the specimen block was cut for histomorphometrical analysis (Figure 3).

### Histomorphometry

Our main interest in this study was the status of the tissues present in the gap after 4 weeks. Unbiased volume density and surface area density of the synthetic bone graft granules was estimated. Systematic uniform random sampling technique combined with isotropic uniform random stereological design used for obtaining unbiased estimates of: 1, volume of tissue and material in the gap, and 2, surface area of the BGE granules in contact with tissue. The surface of the granules was highly irregular and was therefore considered isotropic. A stereological model of a perfect cylinder was used to estimate surface area density of the cylindrical implants (Vestermark 2011).

The 6-mm block specimens were fixed and dehydrated in graded ethanol (70–100%) prior to cold embedding in poly(methylmethacrylate) (Erben 1997). Then they were sectioned, with orientation perpendicular to the axis of the implant. This produced 10–15, 125-µm thick sections per implant with an average distance of 420 µm between sections. Every 2 to 3 sections, 5 sections in total per implant, were sampled for the analysis, and their surfaces were stained with toluidine blue at pH 7. Tissue classification was based on morphology: new bone was a disorganized, dense substance with embedded cells colored dark purple; allograft was a dense substance with empty cell lacunae and clear cement lines colored pale purple. Bone marrow was a cell-rich conglomerate with intervening empty areas from dissolved fat and few scattered thread-like structures. Fibrous tissue was dense, with well-organized bundles of fibers with sparsely intervening small cells. BGE was identified as coarsely profiled shadows.

**Figure 4.**
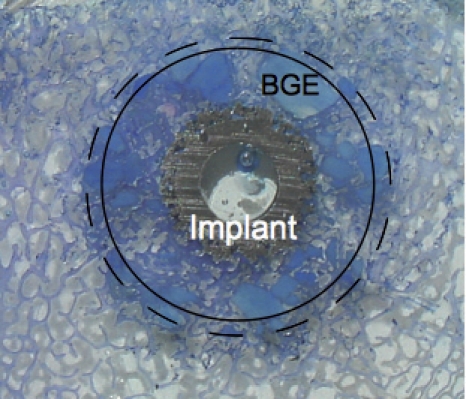
Manually drawn region of interest (ROI) 2.45 mm from the implant surface, shown by the complete circle. BGE, implant with porous coating of beads. The circle with the dashed line shows the approximate position of the drill hole. 1.25× objective.

Osseointegration (gap healing and ongrowth) was estimated in a predefined region of interest (ROI) manually drawn from an applied grid of 2 centralized circles: an inner circle 2.9 mm in diameter to centralize the ROI with regard to the implant, and an outer circle 10.5 mm in diameter to outline the ROI at a distance of 2.45 mm out into the surrounding gap of the implant (Figure 4). The 2 components of osseointegration of the implant were measured by histomorphometry using a stereological system—an Olympus microscope BX50 and Visiopharm Integrator system (NewCast version 3.0.9.0; Visiopharm, Hoersholm, Denmark). Gap healing was defined as volume density fraction of new bone in the ROI. The volume density was measured by counting 276 (SD 52) grid points hitting new bone per specimen. A mean of 1,539 (SD 68) points was counted for each specimen and determined as being either new bone, allograft, fibrous tissue, bone marrow tissue, or BGE.

Ongrowth onto the implant was defined as surface density area fraction of new bone in contact with the implant surface. At interceptions between the surface of the implant and randomly orientated gridlines, the tissue in contact with the implant was determined and counted as being either new bone, allograft, fibrous tissue, bone marrow tissue, or BGE. For each implant, a mean of 756 (SD 54) line intercepts was counted in total and a mean of 107 (SD 54) intercepts was counted for new bone in contact with the implant per specimen.

Ongrowth onto the BGE was also estimated, which was defined as new bone in contact with the surface of BGE granules. At intercepts between the surface of granules and randomly orientated gridlines, the tissue in contact with the granule was determined and counted as being either new bone, allograft, fibrous tissue, or bone marrow tissue. A mean of 833 (SD 121) line intercepts was counted per implant and a mean of 264 (SD 101) intercepts was counted for new bone in contact with BGE.

All estimates were counted at 250× magnification in randomly sampled fields of view in 100% of the ROIs.

A single observer performed the histomorphometrical analysis and was blinded to the treatment of the specimens. The intraobserver variation was determined as coefficient of variation (CV) on double measurements of one randomly selected implant from each treatment arm (Table 2).

**Table 2.** Intraobserver variation presented as coefficient of variation (%) for all parameters counted

	New bone	Allograft	Fibrous tissue	BGE
Gap healing	8.7	18	72	11
Ongrowth, implant	15	0.0	9	101
Ongrowth, BGE	3.7	0.0	53	NA

### Mechanical testing

Thawed specimens were tested to failure by axial push-out of the implant on an MTS Bionics Test Machine (Eden Prairie, MN). The test was performed blind and all specimens were evaluated in one session. The specimens were placed on a metal support jig with a 7.4-mm diameter central opening and under a 5.0-mm diameter cylindrical test probe. The implant was centralized over the opening, thereby assuring a distance of 0.7 mm between the implant and the support jig. The direction of loading was from the cortical surface inward. A preload of 0.5 N was applied to standardize contact conditions before initiating loading. The displacement rate was 5 mm/min with a 500-N load cell. Data points for every 10 µm of displacement were entered into an excel spreadsheet. The force-displacement curve and the mechanical parameters were calculated from the spreadsheet (Baas 2008).

The mean length of the specimens was 2.6 (SD 0.2) mm and the mean diameter was 5.8 (SD 0.1) mm. Individual specimen lengths were used to normalize push-out parameters. Ultimate shear strength (Pa) was determined from the maximal force applied until failure of the bone-implant interface (determined by a sudden drop in force applied to the implant). Apparent shear stiffness (Pa/mm) was obtained from the slope of the linear section of the load-deformation curve. Total energy absorption (in J/m2) was calculated from the area beneath the load-deformation curve until failure (Soballe 1993, Baas 2008).

1 pair of samples had to be excluded from the mechanical test because one of the implants had been misplaced during surgery, resulting in the superficial part (the part used for mechanical testing) not having bone coverage on a small part of the circumference.

### Statistics

Statistical analysis was performed using Intercooled STATA 10.0 software. In this paired study, the data of the differences between treatment arms were not normally distributed for all variables. The data were therefore transformed by natural logarithm (ln) and found to be normally distributed on the ln scale. An absolute difference between the ln of a pair of data equals the ln of the ratio within the pair (Bland and Altman 1996). 2-tailed p-values below 0.05 were considered statistically significant. Results are presented as medians of relative differences between the paired data. The 95% confidence intervals (CIs) were obtained by back transformation of ln-transformed data unless otherwise stated. Calculations of CV% for each variable were done via calculations of transformed mean and transformed standard deviation (SD) (Aitchison 1957).

## Results

All the histological parameters with an intraobserver CV of less than 20% were statistically tested (Table 2).

**Figure 5.**
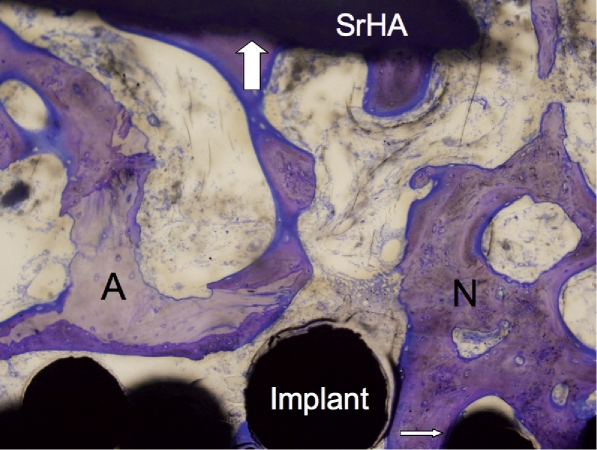
The implant-bone interface showing gap healing and ongrowth of bone onto the implant and SrHA. New bone (N), preserved allograft (A), ongrowth onto the implant (thin arrow), and ongrowth onto the SrHA (thick arrow). 10× objective.

**Figure 6.**
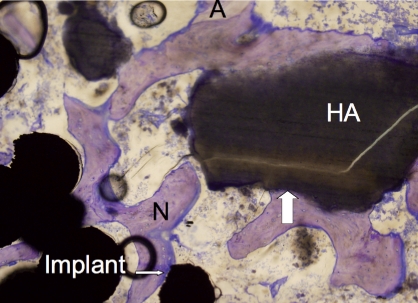
The implant-bone interface showing gap healing and ongrowth of bone onto the implant and HA. New bone (N), preserved allograft (A), ongrowth onto the implant (thin arrow), and ongrowth onto the HA (thick arrow). 10× objective.

**Figure 7.**
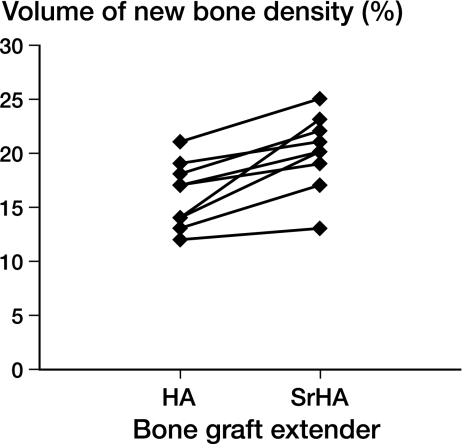
Paired plot of gap healing. The difference in ratio was 1.21 (1.1–1.4); p = 0.003.

**Figure 8.**
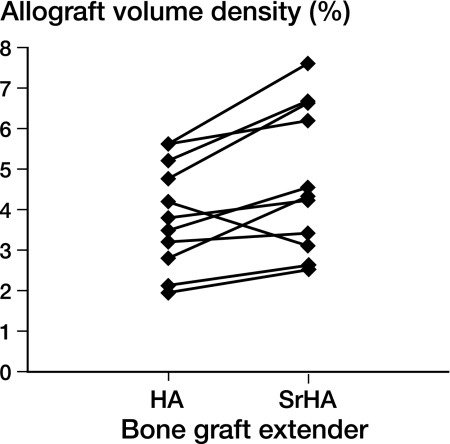
Paired plot of preserved allograft. The difference in ratio was 1.18 (1.02–1.4); p = 0.03.

In the gap, strontium-doped hydroxyapatite (SrHA) induced a statistically significant 21% (CI: 9–35) increase in new bone and 18% (CI: 2–37) more allograft was present compared to HA BGE (Figures 5 to 8). The volume of the BGE was the same for the two treatment arms regardless of strontium doping (–0.2% (CI: –14 to 11)). A statistically significantly greater fraction of the surface area of the SrHA BGE material was also in contact with new bone compared to HA BGE (Figures 5 and 6 and Table 3).

**Table 3.** Ongrowth of bone onto implant and BGE. Histomorphometrical results of surface area fractions of the implant and BGE presented as mean values in per cent with 95% CI in parentheses. The difference between the 2 treatment arms is presented as a median ratio (95% CI). Variation in the data is presented as CV%

	Treatment arms				
	SrHA	HA	Ratio	CV%	p-value
Implant					
New bone	15 (10–21)	13 (7.9–18)	1.25 (0.86–1.8)	56	0.2
Allograft	0	0	NA	NA	NA
Fibrous tissue	27 (9.1–45)	29 (11–46)	0.59 (0.18–1.9)	309	0.3
BGE					
New bone	36 (30–41)	28 (18–37)	1.39 (1.1–1.8)	41	0.03
Allograft	0	0	NA	NA	NA

On the surface of the implant, no statistically significant difference between treatment arms was found for the area fraction of new bone and fibrous tissue in contact with the implant surface. Differences were observed for both parameters in favor of improved osseointegration, but the differences could not be verified statistically at the 5% significance level chosen for this study (Table 3). The same overall results of statistically non-significant improvement were also found for the mechanical parameters ultimate shear strength and total energy absorption. The apparent shear stiffness was the same for the 2 treatment arms regardless of the strontium doping (Table 4).

**Table 4.** Results of the mechanical push-out test presented as mean values with 95% CI in parentheses. The difference between the two treatment arms is presented as median ratio with 95% CI in parentheses. Variation in the data is presented as CV%

	Treatment arms				
	SrHA	HA	Ratio	CV%	p-value
Ultimate shear strength, MPa	2.3 (1.3–3.2)	2.1 (0.76–3.5)	1.25 (0.74–2.1)	75	0.4
Apparent shear stiffness, MPa/mm	12 (7.1–17)	14 (5–23)	1 (0.57–1.7)	83	1
Total energy absorption, kJ/m^2^	415 (211–619)	310 (87–532)	1.62 (0.93–2.8)	82	0.08

## Discussion

Strontium-doped hydroxyapatite (SrHA) as a bone graft extender (BGE) improved gap healing, preserved more of the impacted, morselized allograft, and increased ongrowth of bone onto the BGE. This was statistically evident in the the present study of a Ti implant sorrounded by allograft mixed with hydroxyapatite (HA) as a BGE with or without strontium doping. Type-1 error cannot be ruled out due to the multiple comparisons. We did not prove the main hypothesis that strontium doping of the BGE improves histological and mechanical implant fixation. The unexpectedly high CV of above 30% for the non-significant differences between the treatment arms may have prevented us from detecting a real difference induced by strontium doping of the BGE (type-2 error). Thus, the high CV of 56% for ongrowth of bone onto the implant might have caused the observed increase in bone ongrowth by strontium to be non-significant. The possibility of type-2 error is supported by the statistically significantly increased gap healing and non-significant decrease in fibrous tissue in contact with the implant. Ultimate shear strength and total energy absorption were increased, but not statistically significantly so. While not all significant, all the variables indicated that there was improved histological and mechanical implant fixation. Overall, we interpret these non-significant results as being inconclusive.

We assume that strontium was indeed released from the strontium hydroxyapatite (Christoffersen et al. 1997). Since all other variables were constant, strontium most likely induced the effects that were evident statistically in this study.

Strontium is believed to simulate a homeostatic local hypercalcemia. The reason is that strontium, as also for calcium, is believed to stimulate the calcium-sensing receptor CaSR (Fromigue et al. 2009). The receptor is situated in the membrane of cells of the osteoblast cell line and affects RANKL production and signaling to osteoclasts (Brennan et al. 2009). With regard to osteoclasts, strontium is known to reduce their proliferation and differentiation and it may even induce apoptosis of osteoclasts, which generally reduces resorption of bone (Buehler et al. 2001, Bonnelye et al. 2008). Strontium is also known to enhance the proliferation and differentiation of osteoblasts, which leads to a larger pool of active osteoblasts and thus to an increase in new bone formation (Canalis et al. 1996, Arlot et al. 2007, Atkins et al. 2009).

In this study, these effects of SrHA had two important consequences. Firstly, SrHA increased the healing of the gap surrounding the implant, which was evident from the increased volume of new bone in the gap. Secondly, the resorption of the allograft was delayed when mixed with SrHA, which was evident from the increased volume of allograft in the gap after 4 weeks.

We used dogs because their bone shows great resemblances to human bone. Bone turnover in dogs is higher than in humans, so perhaps a greater response to a stimulus such as strontium would be observed in dogs (Kimmel and Jee 1982, Shaw et al. 1994, Aerssens et al. 1998).

We chose BGE granules with a diameter of 0.6–2.0 mm because this is recommended for clinical settings (Hing 2005). Then, during the mechanical push-out test, we observed that the size of some of the bone graft extender granules was close to the full height of the specimen block. So depending on the position and orientation of large granules of up to 2 mm in diameter, situated in close proximity to the implant, this could either have weakened or strengthened the mechanical properties of the interface in this specific model. In contrast, in a typical clinical setting the height of the gap is greater than the 2.6 mm of the specimen blocks that were subjected to mechanical testing of this study, so the relative size of granules would be smaller and of less importance for the mechanical properties of the interface. The issue of granule size may have contributed to the large variation in the data from the mechanical testing. The exclusion of 1 sample pair weakened the power of the mechanical test, which may have exacerbated the influence of the large variation in data to exceed the expected CVdiff = 30% that was entered in the initial calculation of sample size.

In summary, Ti-alloy implants can become osseointegrated when surrounded by this particular bone graft extender (BGE) of precipitated HA mixed with allograft. This allowed us to investigate whether doping of the bone graft extender with 5% strontium would have any effect on the osseointegration of the implant. The new finding of our study was that histologically, strontium doping of the BGE statistically significantly regulated the imbalance of rapid allograft resorption and relatively slow formation of new bone in the gap. Thus, strontium doping of bone graft extender can be beneficial when an allograft is used during joint replacement surgery. The statistical power of our study was low due to the larger than expected variation in data and because of the loss of 1 sample pair in the mechanical testing. Further investigation is needed to confirm our findings and to determine whether strontium doping of the BGE can improve the full osseointegration and mechanical fixation of allografted implants.
